# Marrying microfluidics and microwells for parallel, high-throughput single-cell genomics

**DOI:** 10.1186/s13059-015-0695-0

**Published:** 2015-06-20

**Authors:** Marc H. Wadsworth, Travis K. Hughes, Alex K. Shalek

**Affiliations:** Department of Chemistry, Massachusetts Institute of Technology, Cambridge, MA USA; Institute for Medical Engineering & Science, Massachusetts Institute of Technology, Cambridge, MA 02139 USA; Broad Institute of MIT and Harvard, Cambridge, MA 02142 USA; Ragon Institute of MGH, MIT and Harvard, Cambridge, MA 02115 USA; Division of Health Sciences and Technology, Harvard Medical School, Boston, MA USA; Immunology, MGH, Boston, MA 02114 USA

## Abstract

An innovative, microwell-based platform for single-cell RNA sequencing (RNA-seq) combines cost efficiency, scalability and parallelizability, and will enable many new avenues of biological inquiry.

See related Research article: http://dx.doi.org/10.1186/s13059-015-0684-3

Over the past several years, it has become increasingly clear that there can be substantial variability in the behaviors of cells once deemed to be identical. This realization has brought about a renewed interest in defining cellular phenotypes and their variation, and in examining how these change under different biological contexts. To achieve this, approaches are needed that can deeply profile individual cells across diverse experimental conditions. Now, by marrying recent advances in molecular biology with microfluidics and microwells, Bose and colleagues present a new platform for achieving this goal [1], opening up exciting new opportunities to explore cells and their heterogeneity.

## Studying cellular heterogeneity with single-cell RNA-seq

Single-cell RNA-seq represents a powerful new approach for investigating the causes and consequences of cellular heterogeneity. As has been demonstrated, co-variation in gene expression across single cells can be used to identify, genome-wide, distinct cell states and cellular circuits, as well as their molecular markers and drivers (‘extrinsic factors’) [2–8]. However, identifying such structure can be difficult as the utility of any one single-cell genomic profile is limited by technical artifacts in sample preparation (‘technical noise’) and the stochasticity associated with biological processes (‘intrinsic noise’) [2].

To overcome these limitations, researchers have focused on increasing their power through examining sets of genes [3–8] or developing approaches for profiling larger numbers of cells [3–8]. In accomplishing the latter, microdevices — including microfluidics [3–5], reverse-emulsion droplets [6, 7] and microwells [8, 9] — have come to play an increasingly important role. These microscale platforms can be tailored to enable easy capture and processing of single cells, reducing labor and costs, while improving efficiency and consistency over conventional single-cell genomic approaches [3–9]. For example, Fluidigm recently released the C_1_ Auto Prep System which couples with an integrated microfluidic chip to enable automated capture and processing of up to 96 single cells in parallel; this facilitated an order-of-magnitude improvement in throughput, with a similar reduction in cost. Leveraging the scale afforded by this platform, researchers have been able to uncover rare immune cell states [3], survey neuronal diversity [4] and assess cellular hierarchy within lung epithelia [5]. Nevertheless, further scaling of this and related systems, necessary for studying increasingly complex cellular ensembles, has been hindered by their reliance on separate, bulky microfluidic channels for delivering processing reagents to each single cell in parallel.

## Achieving scale through early cellular barcoding

Recently, this experimental barrier has been shattered by a new breed of microdevices that re-envision single-cell processing. Collectively, these approaches rely on the fundamental realization that once the genetic material of each single cell being assayed is uniquely barcoded, the cells can be processed in parallel during subsequent steps [6–8]. Significantly, by tagging cellular mRNAs with uniquely barcoded bead-associated primers in reverse-emulsion droplets, studies from Macosko et al. [7] and Klein et al. [6] both increased throughput by up to 100-fold, without a concomitant increase in price. In one of these approaches, Macosko and colleagues used bead-based RNA capture and tagging (‘Drop-Seq’) to analyze the transcriptomes of 44,808 mouse retinal cells, revealing 39 transcriptionally distinct cell populations and their markers [7]; in the other (‘inDrop’), Klein and coworkers used bead-based delivery of barcoded oligo-dT primers to probe population structure and cellular heterogeneity in mouse embryonic stem cell differentiation after withdrawal of leukemia inhibitory factor (LIF) [6].

Importantly, although these exciting systems are capable of robust, high-throughput single-cell processing, they still require extensive support structures (e.g. syringe pumps and controllers) that might limit widespread adoption. An alternative, similar in principle but not in practice, is microwells: here, cells are physically confined in small chambers after settling by gravity [8, 9] rather than through the use of controlled aqueous and oil-phase flows [6, 7]. As a demonstration of the power of such an approach, Fan and colleagues recently unveiled a commercial system that combines microwells with uniquely barcoded beads to rapidly prepare targeted mRNA profiles from approximately 5000 human peripheral blood mononuclear cells (PBMCs) [8]. Nevertheless, their approach, like previous efforts, is serial, and thus best equipped to examine heterogeneity within a single sample, hindering cross-comparisons.

## A parallelizable platform for single-cell genomics

In this issue of *Genome Biology*, Bose and colleagues [1] describe an important technical advance that marries the simplicity of microwells and the early technique of barcoding of droplets with the parallelizability of microfluidics to enable many single cells from multiple different samples or perturbations to be profiled in parallel. The authors’ system consists of five parallel microfluidic channels, each of which contains over 2000 microwells for cell capture and processing. To operate the system, the authors load single cells into microwells by simply flushing in a cell suspension. They then profile single-cell gene expression using either a RNA-printing-based approach or a bead-based capture modality.

The RNA-printing approach is conceptually akin to microengraving [9]: lysis buffer is added and the microwells are quickly pressed against a slide that contains covalently grafted oligo-dT primers. Mature cellular mRNA hybridizes to these oligos and then, after washing, is reverse-transcribed on-chip by flowing appropriate reagents through the device. Expression can then be quantified by hybridizing gene-specific probes to these slide-delimited cDNAs. Importantly, this mode of operation maintains spatial correspondence between cell and well, potentially enabling additional information collected before lysis (e.g. cytokine secretion) [9] to be used in downstream analyses. In the bead-based capture approach, uniquely barcoded oligo-dT beads are co-loaded into the microwells before performing lysis. After mRNA capture, a modified variant of CEL-Seq [10] is performed in which reverse transcription and second-strand synthesis take place on-chip, and other steps are performed off-chip after bead harvest (although, in principle, other amplification strategies are possible).

To demonstrate the utility of their scalable platform, the authors use it in a bead-based capture format to profile approximately 600 cells. In one of their five lanes, they profile U87 human glioma cells; in another, MCF10a human breast cancer cells; and, in the remaining three, a mixture of both. A second, similar experiment compares U87 and the human fibroblast cell line WI-38. Through sequencing the cells prepared during these two runs, the authors detect an average of 635 and 530 genes, respectively, enabling them to clearly distinguish each type from one another. Clearly, there is room for improvement in the number of genes detected and in the fraction of high-quality libraries made using the chip (currently 50 to 70 % of cells); with future optimizations, this strategy could potentially match other high-throughput, but serial, approaches [6, 7]. Most importantly, this method is inexpensive ($0.10 to $0.20 per cell) and can easily be scaled to accommodate additional assay channels and further reduce costs.

## Concluding remarks

In summary, when coupled with additional microfluidic controls, Bose and colleagues’ marriage of microwells and microfluidics has the potential to usher in a new era for high-throughput single-cell genomics, where gene-expression profiles of thousands of single cells, from the same or different experimental conditions, can be rapidly profiled and compared.

## Abbreviations

LIF: Leukemia inhibitory factor
PBMC: Peripheral blood mononuclear cell
RNA-seq: RNA sequencing
